# Benzylamine and Thenylamine Derived Drugs Induce Apoptosis and Reduce Proliferation, Migration and Metastasis Formation in Melanoma Cells

**DOI:** 10.3389/fonc.2018.00328

**Published:** 2018-08-23

**Authors:** Marina Mojena, Adrián Povo-Retana, Silvia González-Ramos, Victoria Fernández-García, Javier Regadera, Arturo Zazpe, Inés Artaiz, Paloma Martín-Sanz, Francisco Ledo, Lisardo Boscá

**Affiliations:** ^1^Instituto de Investigaciones Biomédicas Alberto Sols (CSIC-UAM), Madrid, Spain; ^2^Centro de Investigación Biomédica en Red de Enfermedades Cardiovasculares y Hepáticas y Digestivas, ISC III, Madrid, Spain; ^3^Departamento de Anatomía, Histología y Neurociencia, Facultad de Medicina, Universidad Autónoma de Madrid, Madrid, Spain; ^4^R&D+i Department Faes-Farma, Avda Autonomía, Leioa, Spain

**Keywords:** melanoma, cytotoxicity, chemotherapy, cellular lines, animal models, metastasis, apoptosis

## Abstract

Melanomas are heterogeneous and aggressive tumors, and one of the worse in prognosis. Melanoma subtypes follow distinct pathways until terminal oncogenic transformation. Here, we have evaluated a series of molecules that exhibit potent cytotoxic effects over the murine and human melanoma cell lines B16F10 and MalMe-3M, respectively, both *ex vivo* and in animals carrying these melanoma cells. *Ex vivo* mechanistic studies on molecular targets involved in melanoma growth, migration and viability were evaluated in cultured cells treated with these drugs which exhibited potent proapoptotic and cytotoxic effects and reduced cell migration. These drugs altered the Wnt/β-catenin pathway, which is important for the oncogenic phenotype of melanoma cells. In *in vivo* experiments, male C57BL/6 or nude mice were injected with melanoma cells that rapidly expanded in these animals and, in some cases were able to form metastasis in lungs. Treatment with anti-tumor drugs derived from benzylamine and 2-thiophenemethylamine (F10503LO1 and related compounds) significantly attenuated tumor growth, impaired cell migration, and reduced the metastatic activity. Several protocols of administration were applied, all of them leading to significant reduction in the tumor size and enhanced animal survival. Tumor cells carrying a luciferase transgene allowed a time-dependent study on the progression of the tumor. Molecular analysis of the pathways modified by F10503LO1 and related compounds defined the main relevant targets for tumor regression: the activation of pro-apoptotic and anti-proliferative routes. These data might provide the proof-of-principle and rationale for its further clinical evaluation.

## Introduction

Metastatic melanoma is one of the most therapeutically difficult cancers to be treated, mainly at advanced stages of diagnosis. The incidence of metastatic melanoma has been increasing around the World over the past decades, and death rates rose faster than for other cancers, being melanoma one of the worse in prognosis (1–3). In fact, the mean overall survival of melanoma patients with unresectable distant metastases remains to be less than 1 year (4). Clinical management of melanoma patients also represents a clinical challenge because the lack of contrasted protocols (5–7). This is in addition to the absence of reliable biomarkers identifying groups of patients who could benefit from more specific treatments (8). Many patients are excluded from novel therapies only because of fast high-speed progression of the disease before a clinical positive response can be expected (9, 10). Taken these facts together, consensus exists in the field suggesting that significant improvements of the overall survival rates of melanoma cohorts require an initial fast response to treatment as an inclusion condition (11). Long-term survival could then be achieved by an increased rate of complete responses or long-term stabilization of partial responses in what is defined in the melanoma field as *consolidation phase* (2, 7, 12). The discovery of the frequent BRAF(V600E) mutation in human melanoma tumors offered the first opportunity to develop an oncogene-directed therapy for these patients profiting the use of selective inhibitors of constitutive BRAF activity (11, 13–16). The fact that melanoma cells express activating mutations in BRAF, but not in A-RAF or C-RAF, allowed the development of the small-molecule drug PLX4032, an orally available and well-tolerated selective BRAF inhibitor. Clinical trials demonstrated its therapeutic value for melanomas carrying the activating BRAF mutation. Due to the RAS/RAF/MEK/ERK pathway deregulation in ca. 90% of malignant melanomas, MEK is a current target in drug development and in clinical trials (11, 13, 17–19). However, dose-limiting side effects are observed, and MEK inhibitors that reduce ERK activation in patients show a low clinical response, probably because MEK inhibition promotes an imbalanced compensatory cell signaling that reduces the therapeutic value of these drugs. Several groups have found that BRAF inhibitor-resistant melanoma cell lines can recover ERK phosphorylation independently of the presence of BRAF inhibitors, and the same remains true for the classic chemotherapeutic drug dacarbazine (DTIC) (11, 13, 17, 18, 20–24). For these reasons, the development of novel small molecules that could counteract resistance mechanisms constitutes a first line of research in the melanoma field. Progress in molecular-targeted melanoma therapies have shown significant successful responses in the reduction of tumor size and increased survival in patients (4, 11, 13, 18, 20, 22, 24–27).

In this work, we analyzed the effect of a series of benzylamine/2-thiophenemethylamine (thenylamine)-derived compounds, being F10503LO1 the lead molecule, which exhibited antitumoral activity over a panel of melanoma tumors (NCI-60 human tumor cell lines screen). These drugs have been assayed in different human and rodent cell lines, from hepatoma to leukemia, with consistent results on growth arrest and induction of apoptosis/necrosis in tumor cells. The target of choice was the very aggressive murine melanoma B16F10 and the human melanoma MalMe-3M cell line. Interestingly, both tumor cell lines express the wild type forms of BRAF and p53, offering the possibility to be used as targets for alternative drugs for the treatment of melanoma cells with activating mutations of the BRAF and Ras oncogenes. Our data indicate that these molecules exhibit a potent cytotoxic/antiproliferative activity *in vitro* and in animal models bearing the melanoma cells. These results provide the basis for a meticulous study on the dissection of pathways involved in the mechanism of action of these compounds. Indeed, our studies suggest that the metastatic capacity of both aggressive tumors can be impaired after administration of F10503LO1, providing novel strategies in preventing the dissemination of melanoma cells.

## Materials and methods

### Materials

Reagents were from Sigma-Aldrich-Merck (St Louis, MO, USA) or Roche (Darmstadt, Germany). Murine cytokines and TNFα, IL6, and PGE_2_ ELISA kits were obtained from PeproTech (London, UK) and Cayman Chem. (Ann Arbor, MI). Antibodies were from Abcam (Cambridge, UK) or Cell Signaling (Danvers, MA, USA). Dacarbazine (DTIC) was from TEVA (Petaj Tikva, IL). Reagents for electrophoresis were from Bio-Rad (Hercules, CA, USA). Tissue culture dishes were from Falcon (Lincoln Park, NJ, USA), and serum and culture media were from Invitrogen (ThermoFisher, Madrid, Spain).

### Animal care and preparation of macrophages

Male C57BL/6 and athymic nude mice 12 ± 4-week-old were used and housed under 12 h light/dark cycle and food and water was provided *ad libitum*. Animals were treated following directive 2010/63/EU of the European Parliament. Bone marrow derived macrophages (MF) were obtained from male C57BL/6 mice by flushing pelvises, femurs, and tibiae with DMEM. Bone marrow mononuclear phagocytic precursor cells were propagated in suspension by culturing in DMEM containing 10% FBS, 100 U/ml penicillin, 100 mg/l streptomycin, and 0.2 nM recombinant murine M-CSF (PeproTech) in tissue-culture plates. Precursor cells became adherent within 7 days of culture. MF cells were maintained in RPMI 1640 medium supplemented with 10% FBS for 14 h prior to use.

### Preparation of chemotherapeutic molecules (F10503LO1, F21010RS1–benzylamines-and F60472RS1−2-thiophenemethylamine or thenylamine-)

Solid samples (Table 1) were stored in a silica gel container at 4°C, and dissolved in DMSO to prepare 10 mM stock solutions maintained at −20°C. Further dilutions were prepared in PBS and the equivalent amount of DMSO was used as control for administration to the cells (*in vitro* assays) or to the animals. When F10503LO1 was dissolved in N,N′-dimethyl acetamide (DMA) solution (5% vol:vol of DMA in saline-glucose 5% w:vol), this was prepared on a daily basis in pure DMA and then adding the glucose solution until the final volume was reached. Control animals received the maximal amount of DMA solution lacking F10503LO1.

**Table 1:**
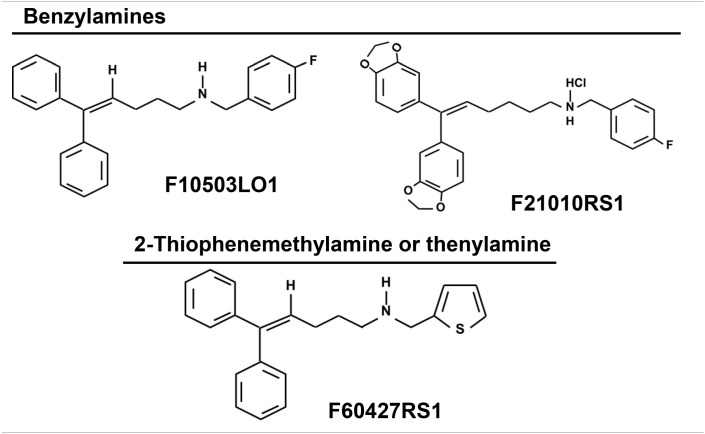
Chemical structure of the drugs.

*The benzylamine derivatives F10503LO1 and F21010RS1 and the 2-thiophenemethylamine derivative (thenylamine) F60427RS1 are represented*.

### *In vivo* administration of melanoma cells

Mice (12 ± 4 weeks-old) were injected 10^6^ cells (200 μl) from the B16F10 melanoma cell line, carrying a luciferase transgene. At the indicated days, F10503LO1 or vehicle (DMSO in PBS as for the drug, or DMA in saline-glucose) were i.p. or i.v. administered. Dacarbazine (DTIC), an alkylating chemotherapeutic agent, was used as reference compound for melanoma treatment and was administered i.p. at 30 mg/kg. For measurement of the tumor growth, animals received i.p. 300 μl of 15 mg/ml luciferine. Luminescence was measured in an IVIS Lumina *in vivo* imaging system (Perkin Elmer, Madrid, ES) under deep anesthesia (isofluorane). Luminescence was recorded each 4 min, with a 1 min capture, repeating the cycle 10 times. The software of the program provides the total area of the luminescence and its quantification.

### Measurement of serum markers

The starting experiments were carried out in male C57/BL6 mice (12 ± 4-wks-old), and an evaluation of the effect of i.p. administration of F10503LO1 on the main biochemical markers of injury was carried out. Animals were challenged with DMSO/PBS (DMSO at the concentration prevailing in the drug administration), or F10503LO1 i.p. at 30 mg/kg at day 1; days 1 to 4; days 1 to 6. Serum was obtained at days 7 and 14 by retroortibal punction. The serum levels of transaminases (GOT and GPT), gamma-glutamyltransferase (GGT), glucose, lactate, triglycerides, cholesterol, uric acid, creatinine and hemoglobin were determined using specific analyte strips (Reflotron; Roche) and measuring the enzyme kinetics in a spectrophotometer. The levels of cholesterol (<100 mg/dl), uric acid (<2 mg/dl), creatinine (<5 mg/dl) and triglycerides (<35 mg/dl) remained indistinguishable between both animal groups at days 7 and 14. These data show a modest impact of F10503LO1 (30 mg/kg; i.p.) on hepatic markers, with an excellent recovery after 1 week without treatment. In addition to this, the absence of changes in creatinine levels, marker of kidney injury, suggested negligible kidney toxicity.

### Evaluation of drug toxicity over myeloid cells

Animals received two consecutive doses of F10503LO1 (30 mg/kg) or vehicle, and the distribution of myeloid cells was determined in blood, bone marrow and spleen on the third day. To analyze leukocyte subpopulations, after euthanizing mice, blood was collected, and spleens and femurs were harvested. All single cell suspensions were subjected to red-blood-cell lysis, incubated with proper dye-conjugated antibodies against CD45, CD115, Ly6G, CD11b, Ly6C, and F4/80 and analyzed in a FACSCanto II flowcytometer (BD). For cell counting, absolute counting beads were used.

### Evaluation of cell viability by flow-cell cytometry

To quantify apoptosis cells were harvested and washed in ice-cold PBS. After centrifugation at 4°C for 5 min, cells were resuspended in annexin V-binding buffer (10 mM HEPES; pH 7.4, 140 mM NaCl, 2.5 mM CaCl_2_). Cells were labeled with annexin V-FITC solution (BD Biosciences, San Jose, CA) and/or propidium iodide (PI; 10 μg/ml) for 15 min at room temperature in the dark. PI is impermeable to living and apoptotic cells, but stains necrotic and apoptotic dying cells with impaired membrane integrity, in contrast to annexin V, which stains early apoptotic cells. Quantification of positive cells was done in a FACSCanto II flowcytometer (BD). Z-VAD-FMK (carbobenzoxy-valyl-alanyl-aspartyl-O-methyl-fluoromethylketone) was used at 20 μM to inhibit caspases.

### Measurement of caspase activity

Cell extracts were prepared at the indicated times and the activity of caspase 3 and caspase 9 were determined using specific commercial fluorimetric kits (Sigma-Aldrich-Merck).

### Measurement of mitochondrial inner membrane potential

To measure the mitochondrial inner membrane potential, cells were incubated at 37°C for 15 min in the presence of 30 nM chloromethyl X-rosamine (CMXRos; ThermoFisher), followed by immediate analysis of fluorochrome incorporation in a FACScanto II flow cytometer. Incubation of the cells with 200 nM of staurosporine was used as a control to induce full mitochondrial-dependent apoptosis as described (28).

### Measurement of accumulation of cytokines, prostaglandin and NO in the cell culture medium

The accumulation of TNF-α, IL-6, and PGE_2_ in the culture medium was measured per triplicate using commercial kits, following the indications of the supplier. Nitric oxide accumulation in the culture medium was measured as nitrite plus nitrate, as previously described (29).

### Histological examination of fixed sections

Anatomopathological analyses were performed using 3 to 5 tumors from each experimental group. Tissue samples were fixed in 10% buffered formalin and embedded in paraffin, and 4-μm sections were prepared. Hematoxylin/eosin stain was used for analysis to assess morphological changes, using a light microscope (Zeiss x20 and x40 images). Examinations of the slides were performed in a blinded fashion.

### Infiltration of B16F10 in tissues

To evaluate metastatic/migratory B16F10 melanoma cells, tissues (lung in particular) were homogenized with 20 mM Hepes, pH 7.4; 100 mM KCl, 5 mM MgCl_2_, 2 mM DTT and luciferease activity was measured in a luminometer using the luciferase assay kit from Promega (WI, USA), following the instructions of the supplier. Usually, 10 μg of protein were assayed in 0.5 ml of reaction mixture.

### Preparation of protein cell extracts

Macrophages total protein extracts were prepared after homogenization in a buffer containing 10 mM Tris-HCl, pH 7.5; 1 mM MgCl_2_, 1 mM EGTA, 10% glycerol, 0.5% CHAPS, 1 mM β-mercaptoethanol and a protease and phosphatase inhibitor cocktail (Sigma). The extracts were vortexed for 30 min at 4°C and after centrifuging for 20 min at 13,000 g, the supernatants were stored at −20°C. When cytosolic and nuclear extracts were prepared, cells were homogenized and processed as previously described (30). Protein levels were determined using Bradford reagent (Bio-Rad).

### Western blotting

Protein extracts were boiled in loading buffer (250 mM Tris-HCl; pH 6.8, 2% SDS, 10% glycerol, and 2% β-mercaptoethanol) and 30 μg of protein were subjected to 8–10% SDS-PAGE electrophoresis gels. Proteins were transferred into polyvinylidene difluoride membranes (GE Healthcare). Membranes were incubated for 1 h with low-fat milk powder (5%) in PBS containing 0.1% Tween-20. Blots were incubated for 2 h or overnight at 4°C with primary antibodies at the dilutions recommended by the suppliers. The blots were developed with ECL Advance protocol (GE Healthcare) and different exposure times were performed for each blot in an ImageQuant analyzer (LAS 500, GE Healthcare) to ensure the linearity of the band intensities. Blots were normalized for lane charge using antibodies against GAPDH.

### RNA isolation and qRT-PCR analysis

RNA was extracted with TRIzol Reagent (ThermoFisher) and reverse transcribed using Transcriptor First Strand cDNA Synthesis Kit for RT-PCR following the indications of the manufacturer (Thermo-Fisher). Real-time PCR was conducted with SYBR Green Master on a MyiQ Real-Time PCR System (Bio-Rad). Primer oligonucleotide sequences are available on request. Validation of amplification efficiency was performed for each pair of primers (29). PCR thermocycling parameters were 95°C for 10 min, 40 cycles of 95°C for 15 s, and 60°C for 1 min. Each sample was run in duplicate and was normalized vs. 36B4. The fold induction (FI) was determined in a ΔΔCt based fold-change calculation.

### Statistical analysis

Unless otherwise stated, data are the mean ± standard deviation. To compare means between two independent samples Mann-Whitney rank sum test was used. Data were analyzed by SPSS for Windows statistical package version v21. Analysis of statistical significance of Kaplan-Meier curves was performed using the Mantel-Cox test. The results were considered significant at *p* < 0.05.

## Results

### Specific effects of new benzylamine- and thenylamine-derived anti-tumor drugs on melanoma cells

**Figure 1:**
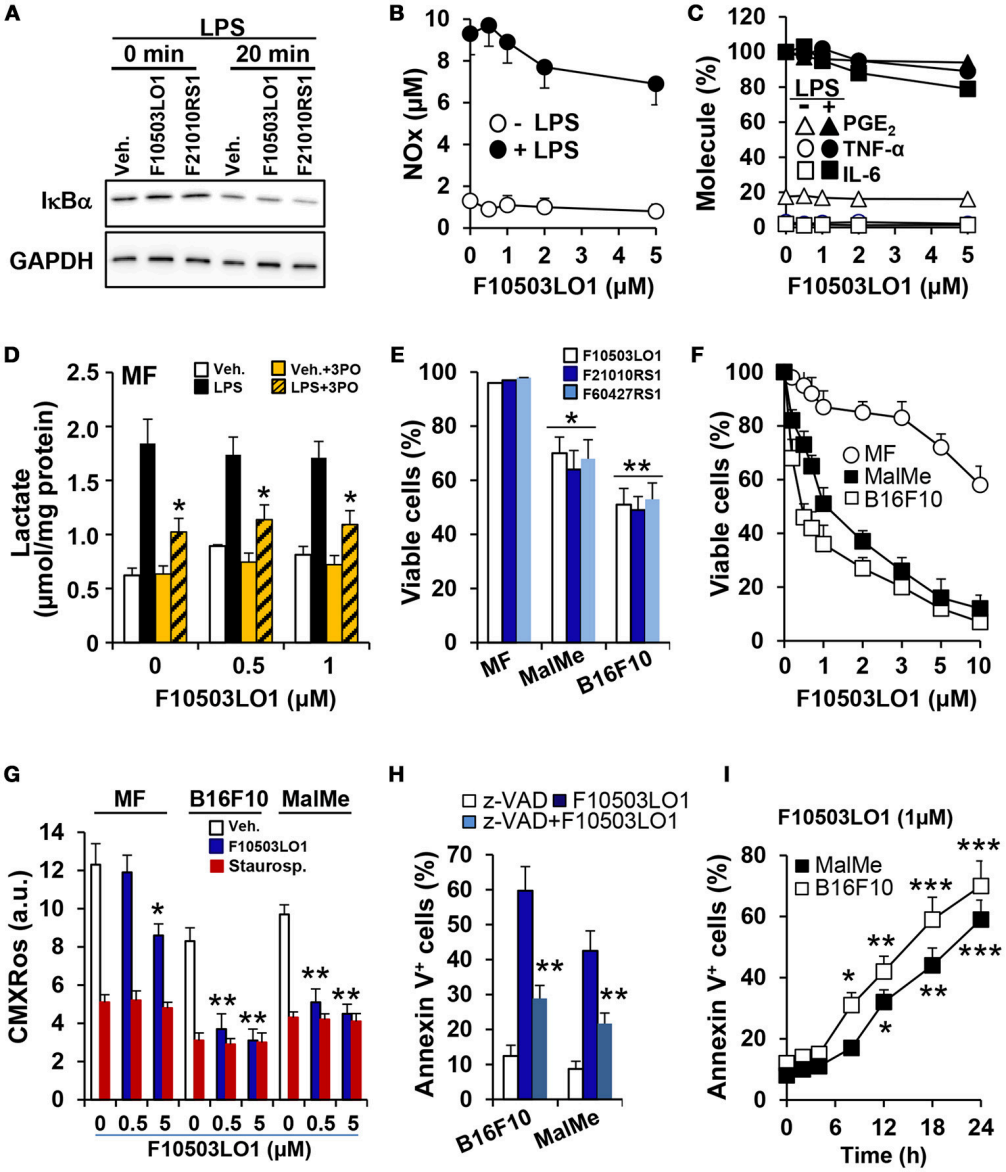
Effect of benzylamine- and thenylamine-derived drugs on cell viability and function. **(A)** Bone marrow derived macrophages (MF) were incubated for 30 min with 1 μM of the indicated molecules or vehicle, followed by challenge with 200 ng/ml of LPS. The degradation of IκBα was evaluated by immunoblot. **(B–D)** The dose-dependent effect vs. F10503LO1 on the accumulation in the culture medium of nitrates and nitrites (in μM), IL6, TNFα, PGE_2_ (expressed as percentage of F10503LO1-untreated cells; 100% corresponds to 12.2, 18.7, and 1.25 ng/ml for IL6, TNFα and PGE_2_, respectively) and lactate were determined after 18 h of treatment with 200 ng/ml of LPS and the PFKFB3 inhibitor 3PO (10 μM). **(E)** The effect of 500 nM of the indicated molecules on the viability of MF and the melanoma cell lines B16F10 and MalMe-3M was determined after 24 h of treatment. **(F)** The dose-dependent effect of F10503LO1 on the viability of MF, B16F10, and MalMe-3M cells was determined at 24 h. **(G)** The mitochondrial inner membrane potential was evaluated after 5 h of treatment with 500 nM F10503LO1 or 200 nM staurosporine (as an inducer of mitochondrial-dependent apoptosis) and measuring the fluorescence (in arbitrary units; a.u.) of 30 nM CMXRos. **(H)** The effect of 10 μM of z-VAD on the apoptosis induced by 500 nM F10503LO1 was determined at 18 h. **(I)** The time-course of the apoptosis induced by 1 μM F10503LO1 was determined at the indicated times. Results show a representative blot **(A)**, or the mean ± SD of three experiments. ^*^P < 0.05; ^**^P < 0.01; ^***^P < 0.005 vs. the corresponding control, untreated cells or macrophages.

The compounds under study were initially characterized by their capacity to interact with adaptor molecules of the NF-κB pathway, a transcription factor involved in oncogenic processes (31), and were tested in the NCI-60 cell line panel that contained 8 melanoma cell lines. Most of these cells were sensitive to the lead drugs used in this study. For this reason, several assays were performed to evaluate the action of the drugs shown in Table 1, on early NF-κB signaling and on cell viability. As Figure 1A shows, the benzylamines F10503LO1 and F21010RS1 failed to modify IκBα levels or the LPS-dependent IκBα degradation in macrophages. Moreover, in murine macrophages stimulated with LPS these drugs minimally altered the accumulation of nitrate plus nitrite (Figure 1B), PGE_2_, TNFα, and IL6 (Figure 1C) or lactate in the culture medium, including the inhibition of the PFKFB3 with the selective inhibitor 3PO (Figure 1D) in LPS stimulated cells. Together, the data indicate that these drugs did not affect the transcription dependent on NF-κB activity. Interestingly, these drugs promoted a loss in viability of human (MalMe-3M) and murine (B16F10) melanoma cell lines, but not in other cells such as resting macrophages (Figure 1E) or resting T and B cells (not shown). Indeed, incubation of melanoma cell lines with F10503LO1 induced a dose-dependent loss in viability, with I_0.5_ *ca*. 500 nM (Figure 1F). This cell death was accompanied by a dose-dependent decrease in the mitochondrial inner membrane potential (Figure 1G), and was partially prevented by the broad caspase inhibitor z-VAD, as deduced by a reduction in the percentage of annexin V-positive cells (Figure 1H). The time course of melanoma apoptotic death is shown in Figure 1I.

### *In vitro* analysis of the effect of benzylamine and thenylamine chemotherapeutic drugs on murine melanoma B16F10 cells

**Figure 2:**
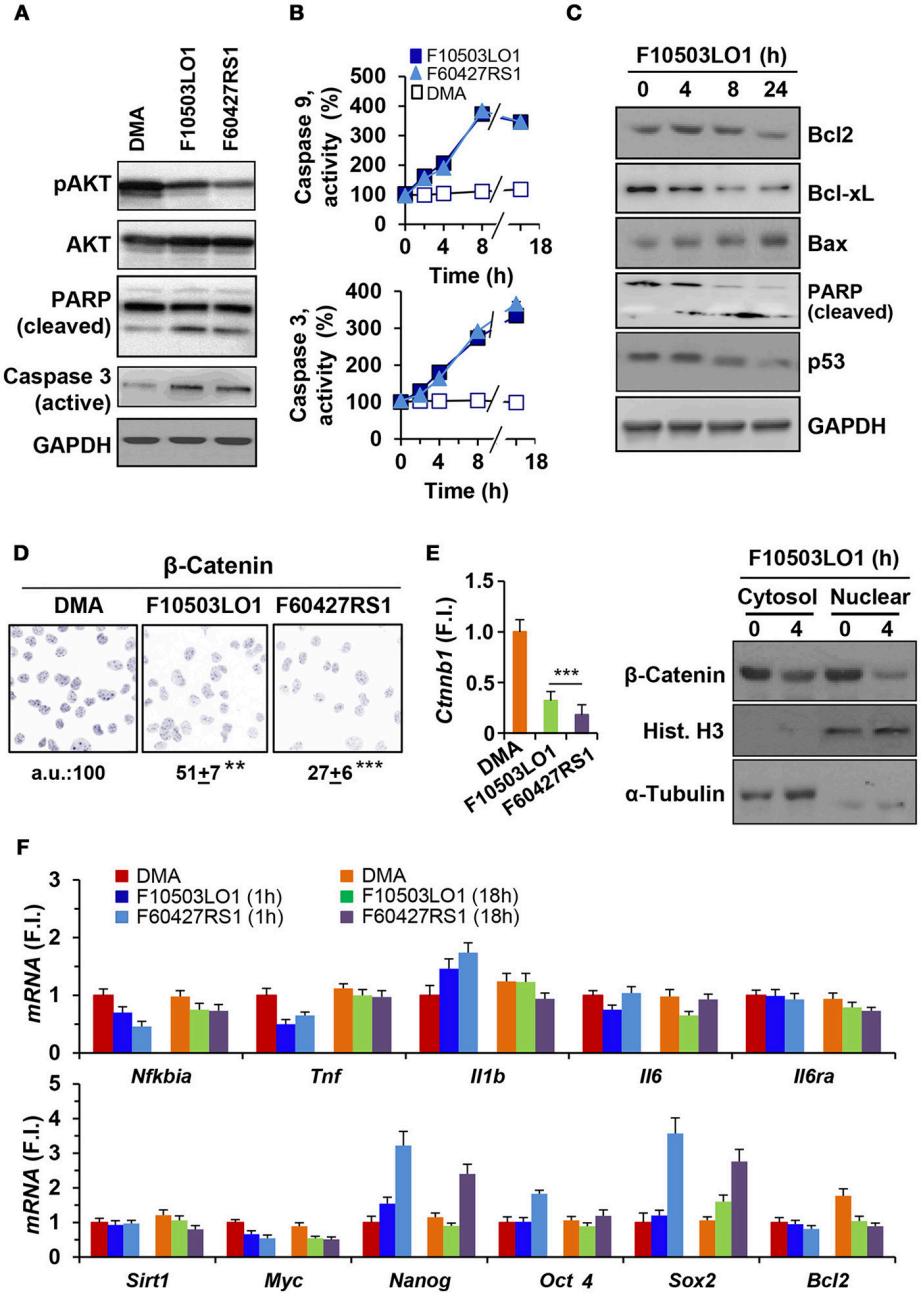
Effect of F10503LO1 and F60427RS1 on apoptosis related proteins, β-catenin and genes related to cell survival. **(A)** B16F10 melanoma cells exhibit constitutive activation of AKT that was inhibited by F10503LO1 and F60427RS1 (1 μM) after 8 h of incubation. PARP and caspase 3 processing were evaluated under these conditions. **(B)** The time course of caspase 9 and caspase 3 activities were determined in cell extracts, using selective fluorescent substrates. **(C)** Time-course of the protein levels of pro-apoptotic and anti-apoptotic genes from B16F10 cells treated with 1 μM of F10503LO1. **(D)** Immunostaining of β-catenin levels in cells treated for 4 h with 200 nM of F10503LO1 and F60427RS1. The intensity of the labeling was referred to the value of untreated cells and expressed in percentage. **(E)** The β-catenin protein levels in the cytosolic and nuclear fractions were determined by immunoblot, as well as the corresponding mRNA levels (*Ctnnb1*) that were determined at 4 h. **(F)** The mRNA levels of the indicated genes were determined at 1 and 18 h after treatment with 200 nM of F10503LO1 and F60427RS1, and expressed as fold-induction (F.I.) vs. cells treated with DMA at 1 h. Results show a representative blot, out of three **(A,C)**. The mean ± SD for the caspases activities **(B)**. A representative staining of β-catenin and the mean±SD of three experiments **(D)**. ^**^P < 0.01; ^***^P < 0.005 vs. DMA condition **(C)**.

B16F10 melanoma cells constitutively exhibit AKT phosphorylation. Treatment with F10503LO1 or F60427RS1 decreased pAKT levels and promoted PARP and caspase 3 activation (Figure 2A). Moreover, measurement of caspase 3 and caspase 9 activities in the cell extracts showed a time-dependent increase in B16F10 cells treated with benzylamine or thenylamine drugs (Figure 2B). These data support the induction of apoptosis in these cells after treatment with these compounds. Indeed, the levels of anti-apoptotic proteins, such as Bcl-xL and to a lesser extent Bcl2, declined and a rise in pro-apoptotic proteins, such as Bax, was observed (Figure 2C). A time-dependent cleavage of PARP was evidenced, with minimal changes in p53 that usually increase in apoptotic cells (Figure 2C). In addition to this, treatment of B16F10 cells with F10503LO1 or F60427RS1 induced a degradation of β-catenin that was also reflected in a downregulation in the corresponding mRNA levels (Figures 2D,E). These changes were accompanied by a decrease in the nuclear content of β-catenin and in the mRNA levels of *Ctnnb1* (Figure 2E). This drop in *Ctnnb1* was quite selective since the mRNA levels of other genes involved in inflammation remained minimally affected (Figure 2F, upper panel). Interestingly, *Myc* and *Bcl2* exhibited a decrease at 1 and 18 h, whereas classic stemness genes, such as *Nanog, Oct4* or *Sox2* increased in cells treated with F60427RS1 (Figure 2F, lower panel), suggesting the existence of specific responses that differentiate the action of benzylamine and thenylamine drugs.

### Melanoma cell migration is inhibited by F10503LO1

**Figure 3:**
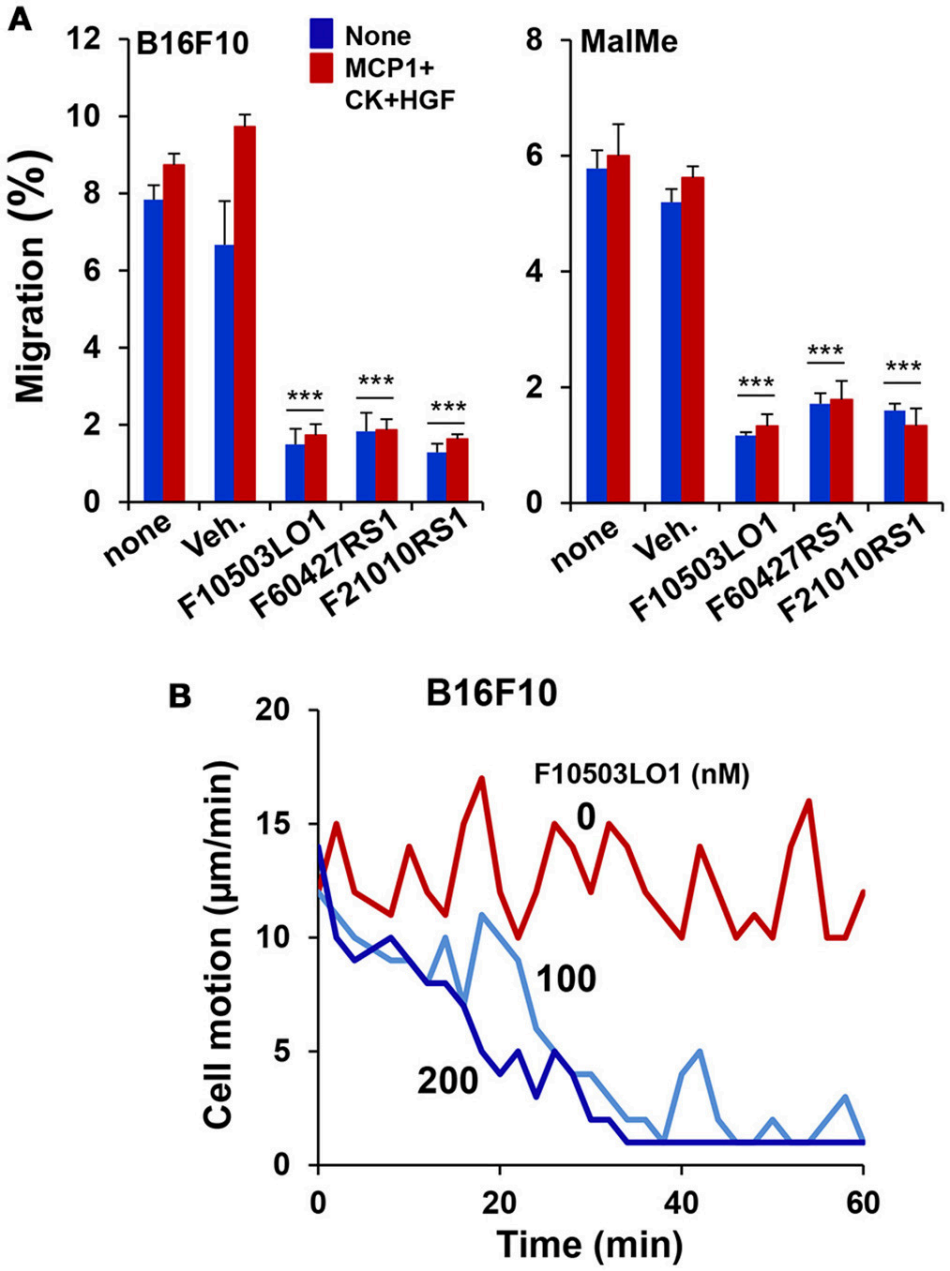
F10503LO1 reduces cell migration and motion. **(A)** B16F10 and MalMe-3M cells were seeded in transwells (uncoated 8 μm porous transwells) according to the manufacturer's instructions. 5 × 10^4^ cells were seeded in the upper chamber and allowed to attach for 2 h in DMEM containing 2%FCS and antibiotics. Non-attached cells were removed after washing with PBS. The lower side of the membrane was cleaned and the transwells were transferred to dishes containing fresh DMEM medium supplemented with 200 nM of the indicated drugs and stimuli (10 ng/ml of MCP1 and HGF). After 3 h, the number of cells present in the lower part of the chamber (including the membrane) were counted and expressed as percentage vs. the total number of cells in the well. **(B)** The motion of the cells was evaluated in a Cell Observer microscope and the corresponding images (Supplementary Videos v1_B16F10, v2_MalMe-3M) were recorded and analyzed. Results show the mean±SD of three experiments per duplicate **(A)**, or a representative experiment of cell motion (**B**, Supplementary Videos). ^***^P < 0.005 vs. the corresponding untreated cells.

In addition to the effects observed on cell viability, 200 nM of F10503LO1, F21010RS1 or F60427RS1 significantly inhibited B16F10 and MalMe-3M cell migration in a transwell assay (Figure 3A). Moreover, melanoma cell motion was also rapidly blocked in a dose-dependent manner suggesting that even doses that are only moderately toxic for these cells decreased their capacity to migrate (Figure 3B, Supplementary Videos v1_B16F10, v2_MalMe-3M).

### *In vivo* effects of F10503LO1

**Figure 4:**
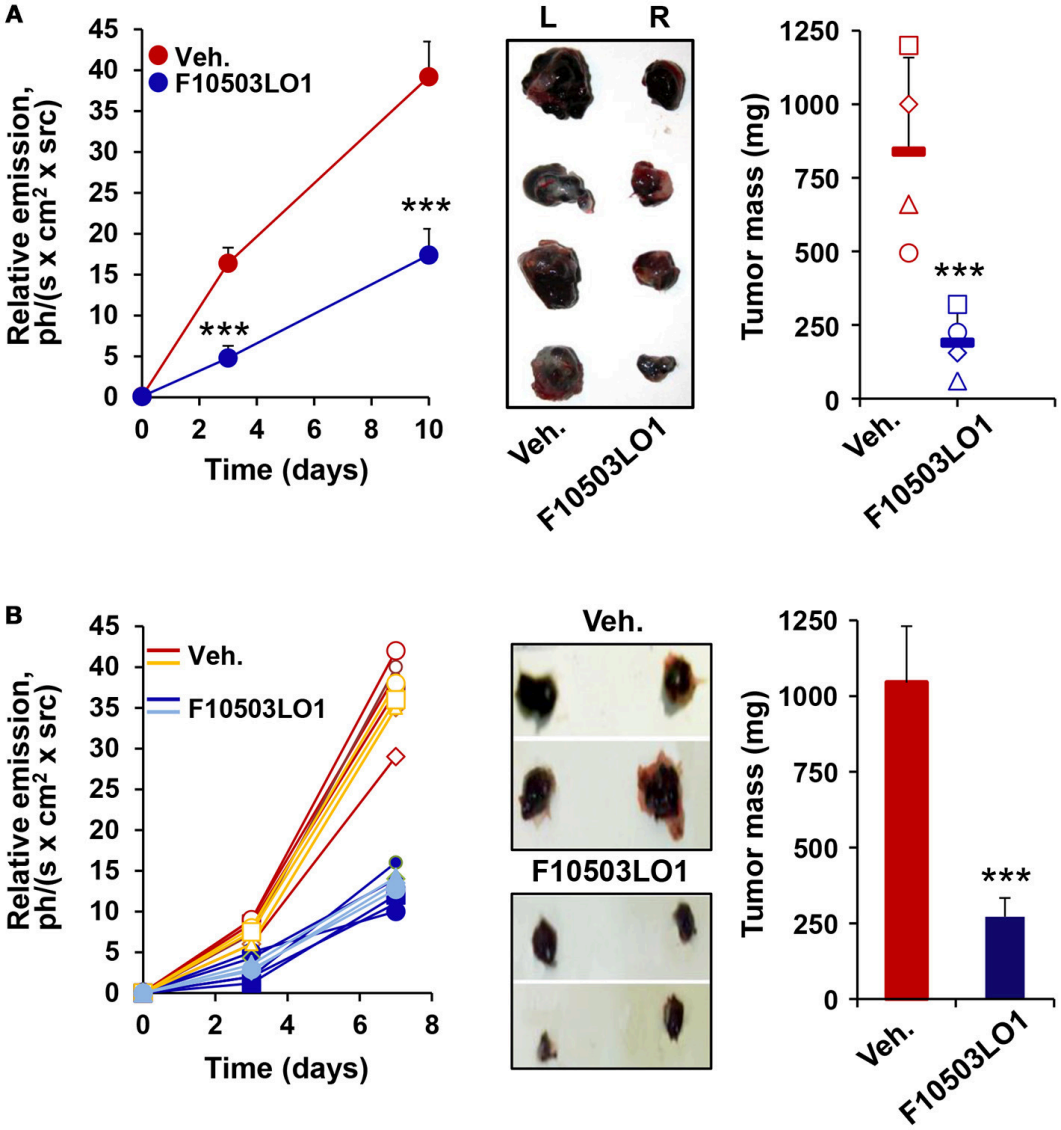
F10503LO1 reduces melanoma tumor growth in mice. **(A)** B16F10 cells carrying a luciferase transgene were treated for 1 h *in vitro* with vehicle (DMSO) or 5 μM F10503LO1. After washing the dishes, 1 × 10^6^ cells were administered to opposite flanks of C57BL/6 mice (*n* = 4). Tumor growth was recorded by the luciferase activity of the B16F10 cells. Tumors were excised off (central panel) and the tumor mass was determined at day 15th. **(B)** B16F10 cells were bilaterally injected (2 × 10^5^ per flank) in nude mice (*n* = 7 per condition), and following 14 days of i.p. treatment with F10503LO1 (30 mg/kg) or vehicle. The luminescence of the tumors was recorded at days 3 and 7 and the tumors were extracted at day 15th (central panel) and weighed. Results show the individual luminescence values **(B)**, and the mean ± SD of the corresponding values. ^***^P < 0.005 vs. the vehicle condition.

**Figure 5:**
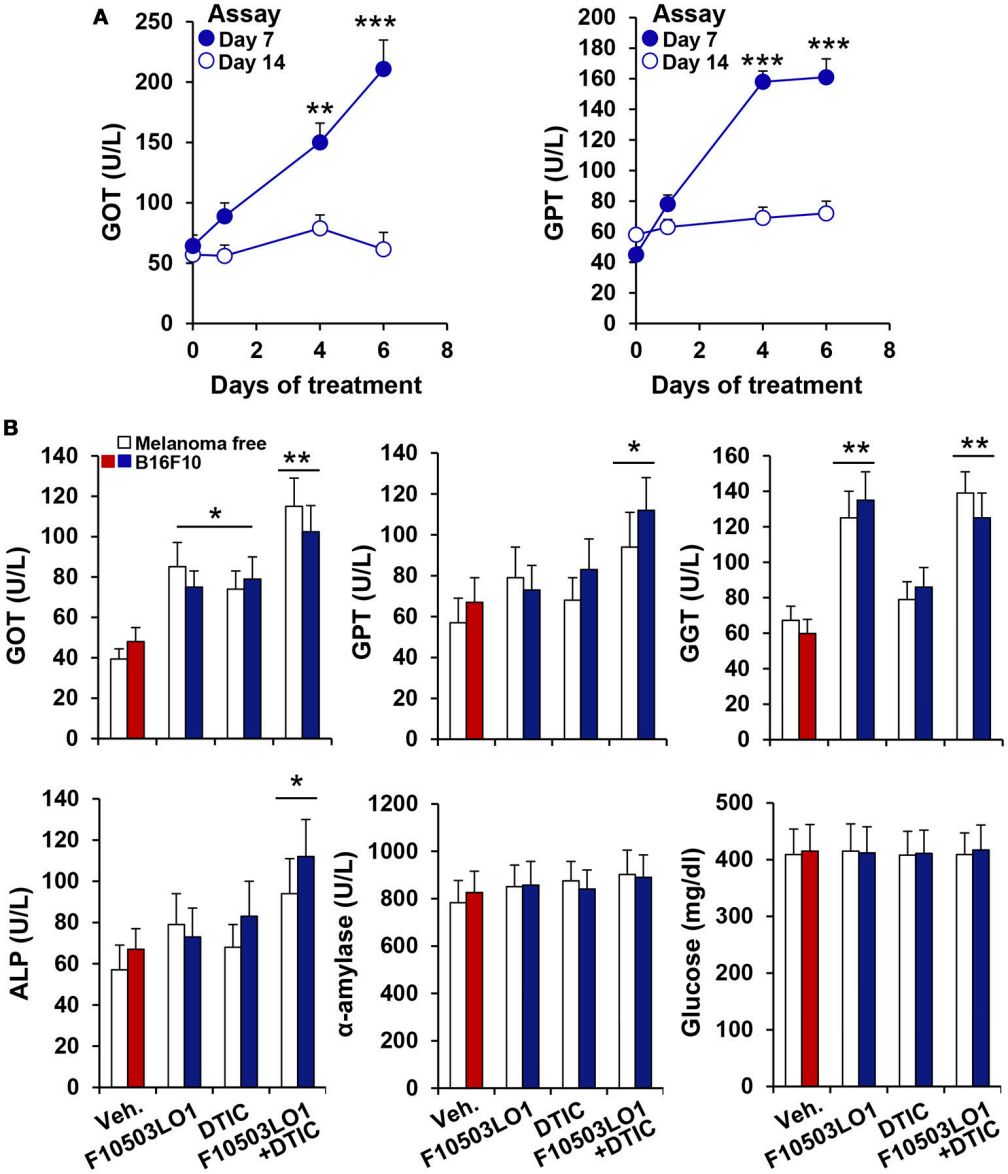
Evaluation of F10503LO1 toxicity in mice. **(A)** Serum levels of GOT and GPT in animals treated i.p. for the indicated periods with 30 mg/kg F10503LO1 (3 for each time: 0, 1, 4, and 6 consecutive days of drug administration). Blood was collected by retroorbital punction at days 7 and 14, and the enzyme activities were measured. **(B)** Animals were injected 1 × 10^6^ B16F10 cells in each flank and F10503LO1 (30 mg/kg), DTIC (30 mg/kg) or both were i.p. administered at days 1 to 5 and 8 to 12. Serum levels of injury markers were determined at day 14. Data are expressed as mean±SD. ^*^P < 0.05; ^**^P < 0.01; ^***^P < 0.005 vs. the corresponding control.

To gain insight on the *in vivo* effects of these drugs, a series of experiments were done in C57BL/6 and in nude mice. As Figure 4A shows, pre-treatment of B16F10 cells for 1 h with 5 μM of F10503 followed by administration in the right flank of mice resulted in a significant inhibition of tumor growth vs. the administration of untreated cells in the contralateral flank, as reflected by *in vivo* luciferase imaging and by the size of the tumors. Subsequently, animals received 2 × 10^5^ B16F10 cells in each flank and 5 h later were i.p. administered 30 mg/kg of F10503LO1 in 200 μl or vehicle. F10503LO1 was provided on a daily basis for 14 days and the *in vivo* luciferase activity was measured at days 3 and 7. At the end of the experiment, tumors were removed, weighted and used for anatomopathological analysis and biochemical processing (Figure 4B). One important point is the evaluation of the broad toxicity of the therapeutic drugs. As Figure 5A shows, F10503LO1 administration during the indicated periods exhibited a moderate rise in serum transaminase levels, and normalization was observed at day 14, suggesting a moderate liver toxicity. The glucose, cholesterol, triglycerides, hemoglobin, creatinine and uric acid concentrations were not affected by the drug (not shown). In addition to this, in animals carrying B16F10 cells and treated i.p. with F10503LO1, the classic chemotherapeutic drug DTIC or combinations of these, the drugs affected moderately transaminases, and other markers, such as gamma-glutamyltranspeptidase (bile duct injury), and alkaline phosphatase (liver and gallbladder injury), but not α-amylase (pancreas injury), glucose and blood lipid levels (cholesterol and triglycerides; not shown) or creatinine (kidney injury; not shown), suggesting a low toxicity of F10503LO1 at the doses used (Figure 5B). In line with these results, the analysis of pro-inflammatory cell markers in blood by flow cytometry showed a tendency to reduce circulating leukocyte populations (Supplementary Figure S1). Of note, an emerging role for the spleen in the pharmacokinetics of drugs has been highlighted recently (32). However, the analysis of pro-inflammatory cells in the spleen also revealed a tendency to low content of monocyte, macrophage and neutrophil cell populations (Supplementary Figure S1), thus excluding the involvement of systemic pro-inflammatory profiles in drug response. Furthermore, the analysis of activated inflammatory cells suggests that F10503LO1 may increase the number of CD11b^+^ lymphocytes (Supplementary Figure S1), highly susceptible of infiltrating in tumors and improve its prognosis (33).

**Figure 6:**
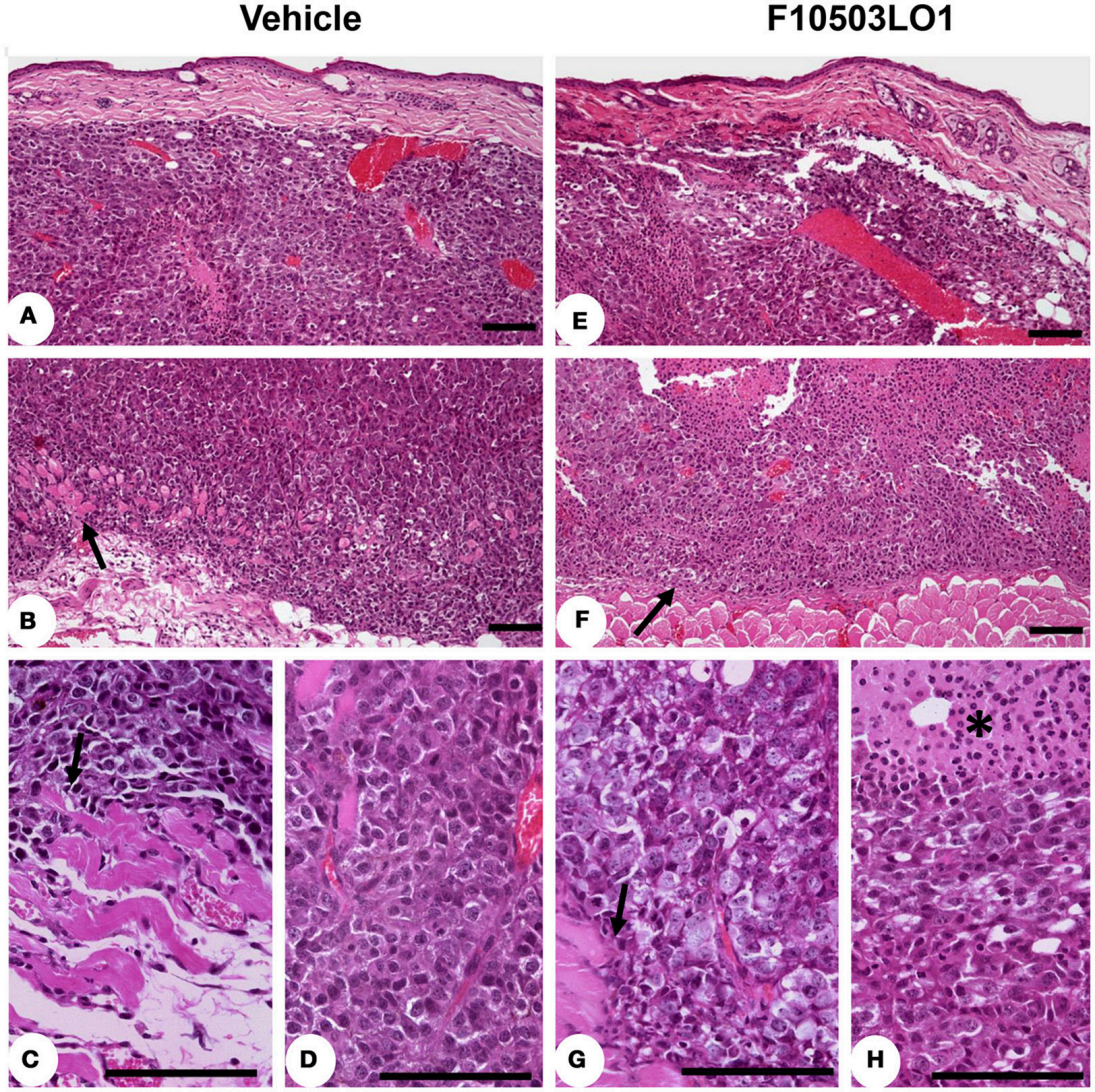
Immunohistochemistry of tumors. Mounted fixed sections of B16F10 melanoma tumors from mice at day 7 and 15 (from Figure 4B). Animals were treated i.p. from day 1 to 6 with vehicle (DMSO) or with 30 mg/kg of F10503LO1. Representative sections of the tumors after hematoxylin-eosin staining. **(A)** diffuse melanoma in dermis reticular; **(B)** infiltration of hypodermis and invasion and destruction of subcutaneous muscular layer (arrow) by melanoma cells; **(C)** the melanoma infiltrating cells produce evident lesions of myocytolysis (arrow); **(D)** pleomorphic melanoma cells with higher mitotic activity in infiltrating tumor; **(E)** F10503 treated melanoma tumor, characterizes by extent invasion of dermis and hypodermis; **(F)** multiple and extends areas of necrosis is seen in treated tumor, and the tumor cells push the muscular striated layer of skin (arrow), but no evident infiltration are observed; **(G)** in detail, the muscular cells sited in the vicinity of melanoma cells not showed apparent myofibril destruction (arrow); **(H)** the melanoma cells showed a clear vacuolated cytoplasm with evident necrotic changes, associated to multiples areas of apoptosis (asterisk). Bar = 100 μm.

In addition to this, fixed samples of experimental melanomas and normal skin were analyzed. Histological studies showed a higher proliferative activity, with an elevated number of mitosis, of the B16F10 melanoma cells. In all cases, an evident invasive capacity of melanoma cells was demonstrated, with invasion of peritumoral adipose tissue and destruction of adjacent muscular striated cells, located in the hypodermis (Figures 6A–D). In the group of melanoma tumors that received administration of F10503LO1, a restricted tumor growth was found. In these treated cases, the infiltrative capacity of tumor cells is low, and the integrity of many skin muscle fascicles are preserved; additionally, apoptotic activity of tumor cells with extends areas of cytolysis and necrosis were observed in melanoma treated tumors (Figures 6E–H).

### *In vivo* studies of F10503LO1 in melanoma-carrying nude mice: comparison with combinations with the chemotherapeutic drug DTIC

**Figure 7:**
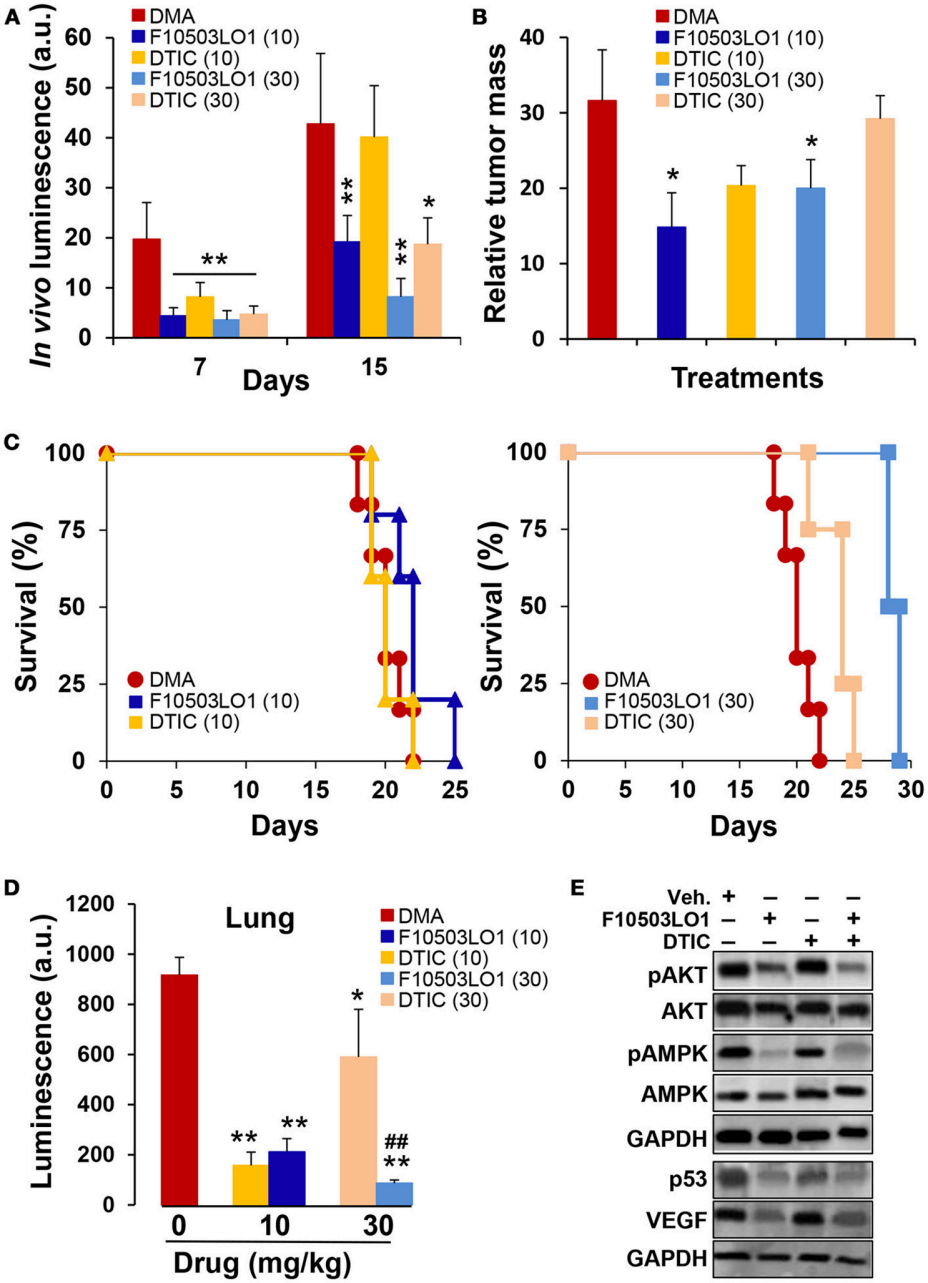
Intraperitoneal administration of F10503LO1 enhances survival of mice-bearing melanoma tumors. **(A)** Nude mice (5 animals for each treatment) received B16F10 cells bilaterally (1 × 10^6^ per flank) and were treated i.p. with the indicated doses of F10503LO1, DTIC (in mg/kg) or vehicle (DMA) at days 4 to 8 and 10 to 15. *In vivo* luminescence lectures were obtained at days 7 and 15 **(A)**. After day 15, animals were maintained untreated and survival was determined. **(B)** Relative tumor mass in dying mice (mg/days of survival). **(C)** Kaplan-Meier plot of animal survival; *P* = 0.0097 (30 mg/kg DTIC vs. DMA); *P* = 0.0022 (30 mg/kg F10503LO1 vs. DMA); *P* = 0.042 (10 mg/kg F10503LO1 vs. DMA). DTIC at 10 mg/kg was not statistically significant vs. DMA. **(D)** Samples of lung from tumor-bearing mice were homogenized and the luciferase activity measured. **(E)** Tumor samples (*n* = 6) were obtained at day 12 and extracts were prepared for Western blot analysis. Data are expressed as mean±SD. ^*^P < 0.05; ^**^P < 0.01 vs. DMA controls; ^##^*P* < 0.01 vs. DTIC at 30 mg/kg.

Nude mice carrying the B16F10 melanoma bilaterally injected were treated i.p. with F10503LO1 or DTIC at the same doses. Drugs were given i.p. at 10 or 30 mg/kg at days 3 to 7 and 10 to 14. After this period, drug administration ceased and animals were kept until death. The tumors were resected and weighed, and several tissues (lung and liver) were excised off and frozen. *In vivo* luminescence was measured at days 7 and 15. As Figures 7A,B shows, both F10503LO1 and DTIC at 30 mg/kg, and F10503LO1 at 10 mg/kg significantly inhibited tumor growth. Animal survival was determined (Figure 7C) and, after animal death, the tumor mass was quantified (Figure 7B). To note that after suppression of drug treatment, tumors expanded in all cases; however, the tumor mass in animals treated with F10503LO1 was significantly lower than in DMA or DTIC-treated animals. In addition to this, samples of liver and lung were homogenized and the luciferase activity was determined as an index of infiltration of B16F10 cells. In the liver, the luminescence was undetectable. However, the lungs exhibited a significant luciferase activity (Figure 7D). Interestingly, animals treated with F10503LO1 that exhibited the maximal survival, showed minimal infiltration in the lung; however, no metastases were evident upon anatomopathological observation (not shown). Tissues obtained at day 12 of treatment were analyzed for the presence of pAKT, pAMPK, VEGF, and p53. As Figure 7E shows, treatment with F10503LO1 decreased AKT and AMPK phosphorylation and p53 and VEGF levels; again, this drug was more efficient than DTIC on the attenuation of these survival, proliferation and angiogenic markers.

**Figure 8:**
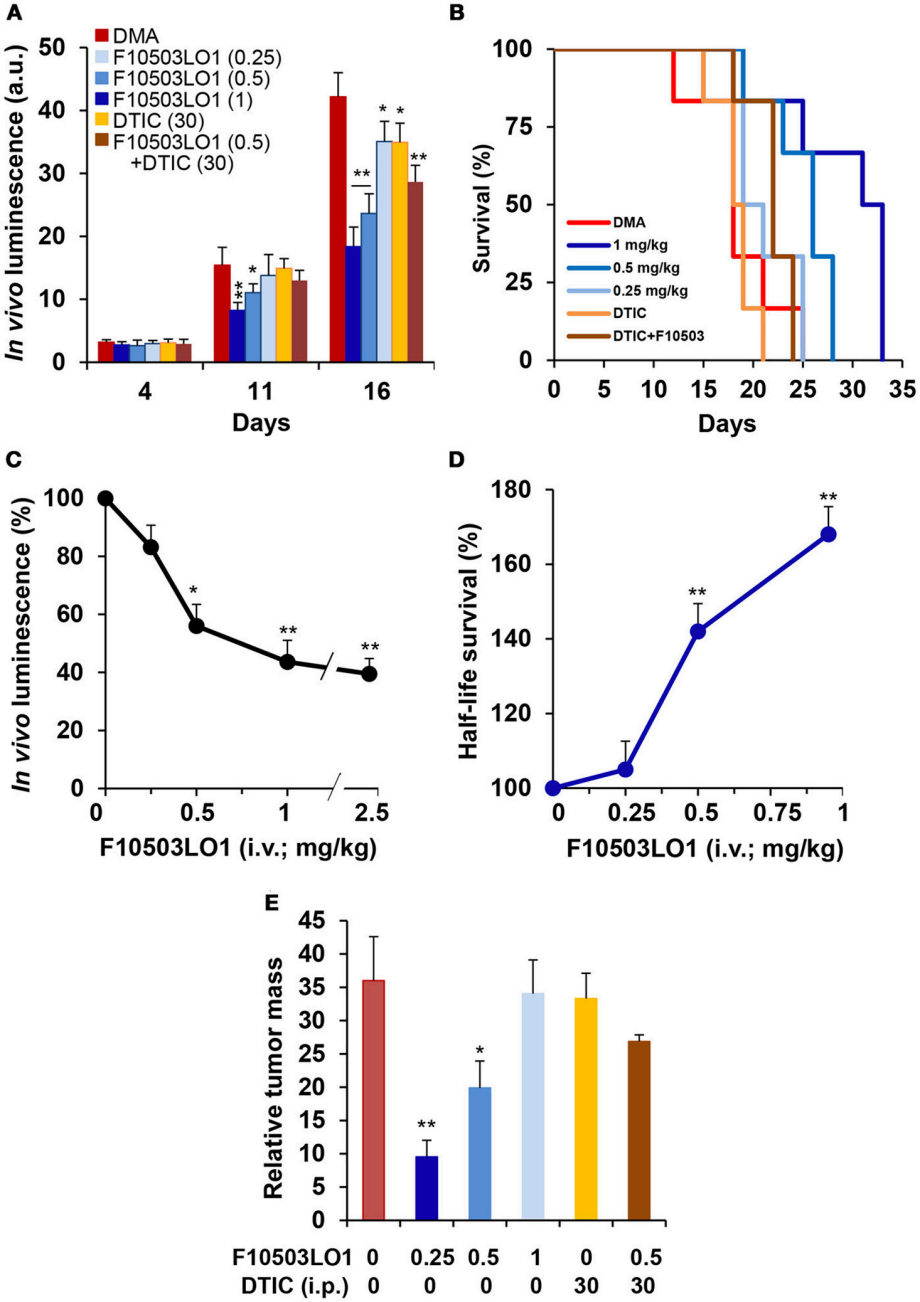
Intravenous administration of F10503LO1 enhances survival in mice-bearing melanoma tumors. **(A)** B16F10 melanoma tumor growth in nude mice treated i.v. with 1, 0.5, 0.25 mg/kg of F10503LO1, i.p. 30 mg/kg DTIC, or i.v. 0.5 mg/kg F10503LO1 and i.p. 30 mg/kg DTIC at days 1, 4, 7, 10, 14, 17, 21, 24, and 28. Each treatment involves 6 mice injected bilaterally with 1 × 10^6^ B16F10 cells. **(B)** Kaplan-Meier survival curves. The statistical significance was; *P* = 0.0091 (0.5 mg/kg vs. DMA); *P* = 0.0089 (1 mg/kg vs. DMA); *P* = 0.379 (DTIC vs. DMA); *P* = 0.9647 (DTIC+F10503LO1 vs. DMA). **(C)** Dose-dependent luminescence curve at day 16. **(D)** Half-life survival curve after F10503LO1 administration. **(E)** Relative tumor mass of the different treatments (mass of the tumor/days of survival). Data are expressed as mean ± SD. ^*^P < 0.05; ^**^P < 0.01 vs. DMA condition.

An additional set of experiments was carried out using 3 different doses of F10503LO1 administered i.v. and in combination with DTIC, given i.p. at 30 mg/kg. F10503LO1 was administered i.v. through the tail vein at 0.25; 0.5, and 1 mg/kg bodyweight at days 1, 4, 7, 10, 14, 17, 21, 24, and 28, following B16F10 bilateral administration. A combination of i.p. DTIC and i.v. 0.5 mg/kg F10503LO1 was included. Luminescence lectures were taken at days 4, 11, and 16. Figure 8A shows the luminescence records for 8–10 tumors (4–5 animals) per each condition, and the Kaplan Meier plot of animal survival (Figure 8B). Figure 8C shows the luminescence associated to the tumors at day 16, including a series of three animals treated i.v. 2.5 mg/kg F10503LO1. Animals treated with 1 mg/kg F10503LO1 exhibited lesser tumor mass at the time of death. Figure 8D shows the increased half-life of the animals vs. the dose of F10503LO1 administered. After animal death, the tumors were excised off and weighted (Figure 8E). These data indicate that F10503LO1 significantly reduced tumor growth; however, the possibility of tumor evasion and metastatic development cannot be excluded as a cause of death. Finally, and supporting previous data, the effect of DTIC on tumor growth was less effective than F10503LO1, and there was a lack of synergism between both drugs under these experimental conditions.

## Discussion

Melanocyte tumorigenesis involves different types of lesions, from benign nevi to malignant melanomas. Because melanocytes are derived from the neural crest and are present in several tissues, a diversity of melanoma phenotypes account for these tumors, which also carry distinct mutations (1, 3, 10, 20, 34). The most common mutated genes are BRAF, p53, NRAS, and KIT, and these mutations use to accumulate in the course of malignization (11, 14, 18, 35). In fact, these mutations occur in different combinations and temporal sequences affecting the activity of genes that regulate key signaling pathways: DNA damage repair, proliferation, cell cycle regulation, cell-specific metabolism, resistance to apoptosis and replicative lifespan among other. In this regard, the area of the discovery and assessment of new biomarkers for melanoma progression is under continuous development (36). Additionally, other factors, such as an enhanced reactive oxygen production appears to be critical in the success for the treatment of melanoma cells that acquired resistance to the BRAF chemotherapy (37). This is one of the main reasons why melanomas have to be attacked combining several chemotherapeutic drugs (3, 18, 23, 38). Indeed, novel drugs are on the pipeline of the pharmaceutical industry. In this work we investigated a series of lead molecules that blocked the interaction between the signaling adaptor p62 and the NF-κB pathway related to tumorigenesis (39), followed by screening on the NCI-60 panel of cancer cells (40). Under these premises, benzylamine and thenylamine-derived molecules emerged as lead candidates for the study of their action on melanoma cells. Interestingly, these drugs, did not affect NF-κB activity in cells such as macrophages, but compromised the viability of human and murine melanoma cells by promoting apoptosis and inhibiting survival pathways and cell migration when used in the 0.5–1 μM range.

Among the assayed molecules, F10503LO1 proved to exhibit a reduced systemic toxicity as reflected by the minor impact on myeloid cell generation in the bone marrow. Administration of F10503LO1 *via* i.p. or i.v. induced only a minor hepatic injury, but did not show alterations in other classic injury-markers associated to kidney, gallbladder and pancreas, nor did it in blood lipid and metabolic markers in C57BL/6 and nude mice. Interestingly enough, control animals recovered normal serum levels of altered injury markers in less than 1 week of cessation of F10503LO1 administration.

*In vitro* effects of F10503LO1 on melanoma cells suggested a potential efficacy in *in vivo* models of melanoma tumorigenesis. In fact, not only did F10503LO1 exhibit cytotoxicity (apoptosis) on B16F10 cells, but it impaired melanoma infiltration and metastases in distal organs (liver, lung) as well. From a molecular point of view, F10503LO1 decreased the content of phospho-AKT, and phospho-AMPK, impaired angiogenesis through a decrease in the intratumor content of VEGF and decreased p53 levels suggesting a specific mechanism leading to a reduced *in vivo* viability of B16F10 cells and tumor dissemination. In addition, the observation that AMPK is dephosphorylated in samples of tumors treated with the drug probably contributes to cancer cell death due to the inability to provide energy substrates to the growing tumor (41). Interestingly, DTIC did not reproduce these effects, nor did it exhibit a significant synergism with F10503LO1 in terms of signaling or tumor growth arrest, prevailing the action of the benzylamine derivative over the DTIC treatment (5, 21). Complementary to these studies, the *in vitro* effects of F10503LO1 and F60427RS1 on melanoma cells well supported the *in vivo* data on tumorigenesis. Both F10503LO1 and F21010RS1 decreased the content of phospho-AKT, at the time that activate PARP and caspase 9 and 3, all mechanisms compatible with the observed loss of viability of the melanoma cells. Interestingly enough, the thenylamine F60427RS1 was as effective as the benzylamines in promoting AKT dephosphorylation, caspase 3/9 activation and inducing apoptosis, but included a rise in acetyl-CoA carboxylase phosphorylation that is frequently associated to an elevation of cytoplasmic calcium. In addition to this, treatment with F10503LO1 or F60427RS1 rapidly downregulate β-catenin levels both in B16F10 and MalMe-3M cells; however, the impact of this pathway in melanoma pathology remains to be controversial (38, 42–44). Regarding the potent effect of the drugs on the Wnt/β-catenin pathway, it should be mentioned that two types of cell surface receptors appear to be involved in its activation: the low density lipoprotein receptor–related proteins 5/6, and the GPCR-coupled Frizzled receptors (44). Indeed, this pathway is activated in many cancer cells leading to dysregulated cell growth and tumorigenesis, at the time that it is mutated in several oncogenic processes, such as melanoma (45). However, due to the diversity in the origins of melanoma, conflictive and opposite views have been proposed regarding the possibility to target the Wnt (more than 19 proteins in this family) and the β-catenin pathways (acting as a coactivator of transcription factors involved in chromatin remodeling). Whereas some authors described that activation of the Wnt/β-catenin pathway is involved in a better prognostic on melanoma metastasis other groups reported opposite results, probably due to the fact that the mutations present in melanoma cells determine the onset of the pathway and the possibility of the effectiveness of immune-regulatory responses in animal models (43, 45–47). This aspect is under study, due to the rapid degradation observed in the β-catenin levels, but we do not know if the β-catenin-dependent transcriptional activity has been fully expressed prior to degradation.

The growth of the melanoma cell line B16F10 implanted in nude mice was reduced after i.v. or i.p. treatment with F10503LO1. In agreement with this, F10503LO1 was able to expand animal survival significantly vs. animals treated with vehicle. This antitumoral activity was dose-dependent and it was observed in almost all the applied administration protocols. F10503LO1 impairs tumor growth at concentrations in the range 0.25–1 mg/kg when administered i.v. twice a week. Administration of 1 mg/kg of F10503LO1 under this protocol extended survival from 25 days in vehicle-treated animals to 33 days in F10503LO1LO1-treated mice. In addition to this, significant reduction in tumor growth was observed in C57/BL6 mice after i.p. treatment, suggesting efficacy for these drugs in the different ways of administration tested. Comparison of the effect of F10503LO1 (i.p. at 30 mg/kg or i.v. at 1 mg/kg) with the reference drug DTIC—an alkylating chemotherapeutic compound—at 30 mg/kg showed a greater effect of F10503LO1 in terms of tumor growth arrest and survival. However, both drugs failed to show any significant synergism under the experimental conditions used in this report.

Anatomopathological analysis of samples of tumors from animals treated with F10503LO1 showed wide areas of cytolysis, necrosis and lesser number of mitotic cells. Advanced tumors from untreated animals exhibited infiltration of the melanoma cells in muscle, adipose tissue and skin at the subcutaneous level, tissues that were more preserved when F10503LO1 was administered. Although no anatomopathological evidence of lung or liver metastases was observed in the different analyzed sections, in whole lung extracts luciferase activity was detected suggesting that some foci were present within the tissue; however, F10503LO1 prevented significantly this metastatic activity as evidenced in the tissue extracts from all analyzed animals. In addition to this, the luciferase activity in lung (indicative of potential metastasis) was significantly lower in animals treated with F10503LO1 when compared to the DTIC counterparts at the highest survival periods.

In summary, the benzylamine and thenylamine derived drugs assayed in this work can be envisaged as an interesting novel molecules for the treatment of melanoma in terms of the efficacy in counteracting the *in vivo* tumor growth of the aggressive murine and human melanoma cell lines assayed, and would provide the proof-of-principle and rationale for further clinical evaluation. Extension of these studies to other human-derived melanoma cells will support this idea, in particular in view of the clear benefits over the action of the classic DTIC treatment. Moreover, the provision additional molecular mechanisms of action for these drugs might help to unravel their relevant targets in the inhibition of tumor growth and promotion of melanoma cell death, alone or in combination with other well established strategies in the field.

## Author contributions

MM, AP-R, and SG-R performed part of the experiments and contributed to the conception, and progress of the work. VF-G performed part of the experiments on flowcytometry. JR performed the immunohistochemistry analysis and discussed the experimental protocols. MM, AZ and IA contributed additional confirmatory experiments and discussed the results. PM-S revised and discussed the manuscript. FL and LB contributed to the conception, design and analysis of the manuscript. AP-R and LB wrote the first draft of the manuscript. All authors contributed to manuscript revision.

### Conflict of interest statement

AZ, IA, and FL were employed by company FAES-FARMA, Spain. The remaining authors declare that the research was conducted in the absence of any commercial or financial relationships that could be construed as a potential conflict of interest.
