# Supplementary material for: Benzylamine and Thenylamine Derived Drugs Induce Apoptosis and Reduce Proliferation, Migration and Metastasis Formation in Melanoma Cells
Source: Front Oncol. 2018 Aug 23;8:328. doi: 10.3389/fonc.2018.00328 (PMC6115490; doi:10.3389/fonc.2018.00328)

Supplementary Material

Preclinical evaluation of new molecules for the treatment of melanoma: Studies in cellular and animal models

Marina Mojena[1#], Adrián Povo-Retana[1#], Silvia González-Ramos[1,2#], Victoria Fernández-García[1], Javier Regadera[3], Arturo Zazpe[4], Inés Artaiz[4], Paloma Martín-Sanz[1,2], Francisco Ledo[4*] and Lisardo Boscá[1,2]

*** Correspondence:** lbosca@iib.uam.es

1 Supplementary Figure

Supplementary Figure S1: Evaluation of F10503LO1-dependent effects of immune cells in mice. Flow cytometry analysis of total leukocytes, activated leukocytes, neutrophils, macrophages and monocyte subsets in bone marrow, blood and spleen from vehicle (n=4) and 30 mg/kg F10503LO1 treated mice (n=3). All myeloid cells were detected based on CD45 expression. Inflammatory leukocytes were discriminated based on the additive expression of CD11b. Classical monocytes were detected based on the expression of Ly6C in $CD115^+$ $CD11b^+$ cells. Macrophages were gated as $F4/80^+$ cells; meanwhile, neutrophils were detected based on their $CD115^-$ $CD11b^+$ $Ly6G^+$ expression. Graphs represent mean ± SEM.

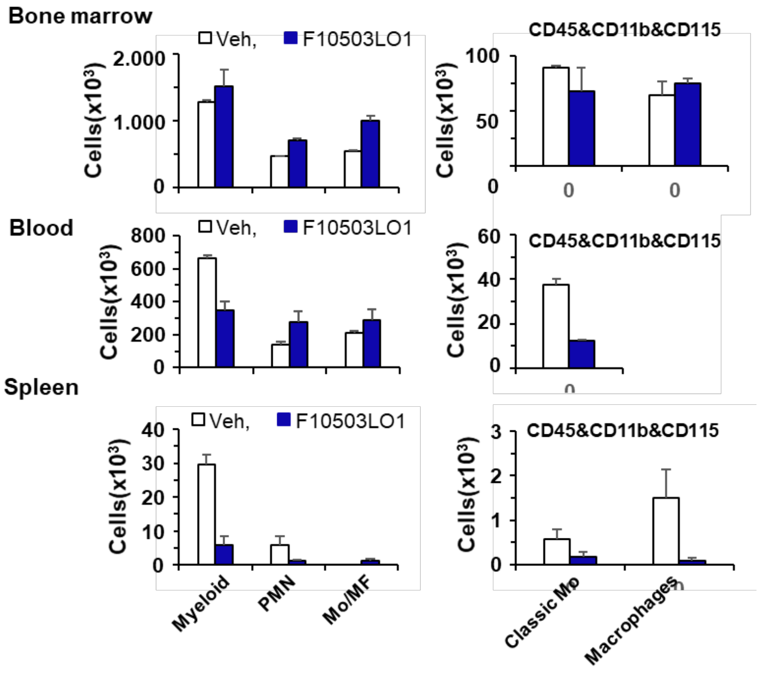

Supplement: Supplementary file 3 [file Image_1.PDF]
